# Supplementary material for: The interactome of intact mitochondria by cross-linking mass spectrometry provides evidence for coexisting respiratory supercomplexes
Source: Mol Cell Proteomics. 2017 Dec 8;17(2):216–32. doi: 10.1074/mcp.RA117.000470 (PMC5795388; doi:10.1074/mcp.RA117.000470)
Supplement: Supplemental Data [file supp_RA117.000470_133922_0_supp_23978_fzffwf.zip › spectra_annotation/mito_DR_spectra_annotation/205-1-8-1-10-1.pdf]

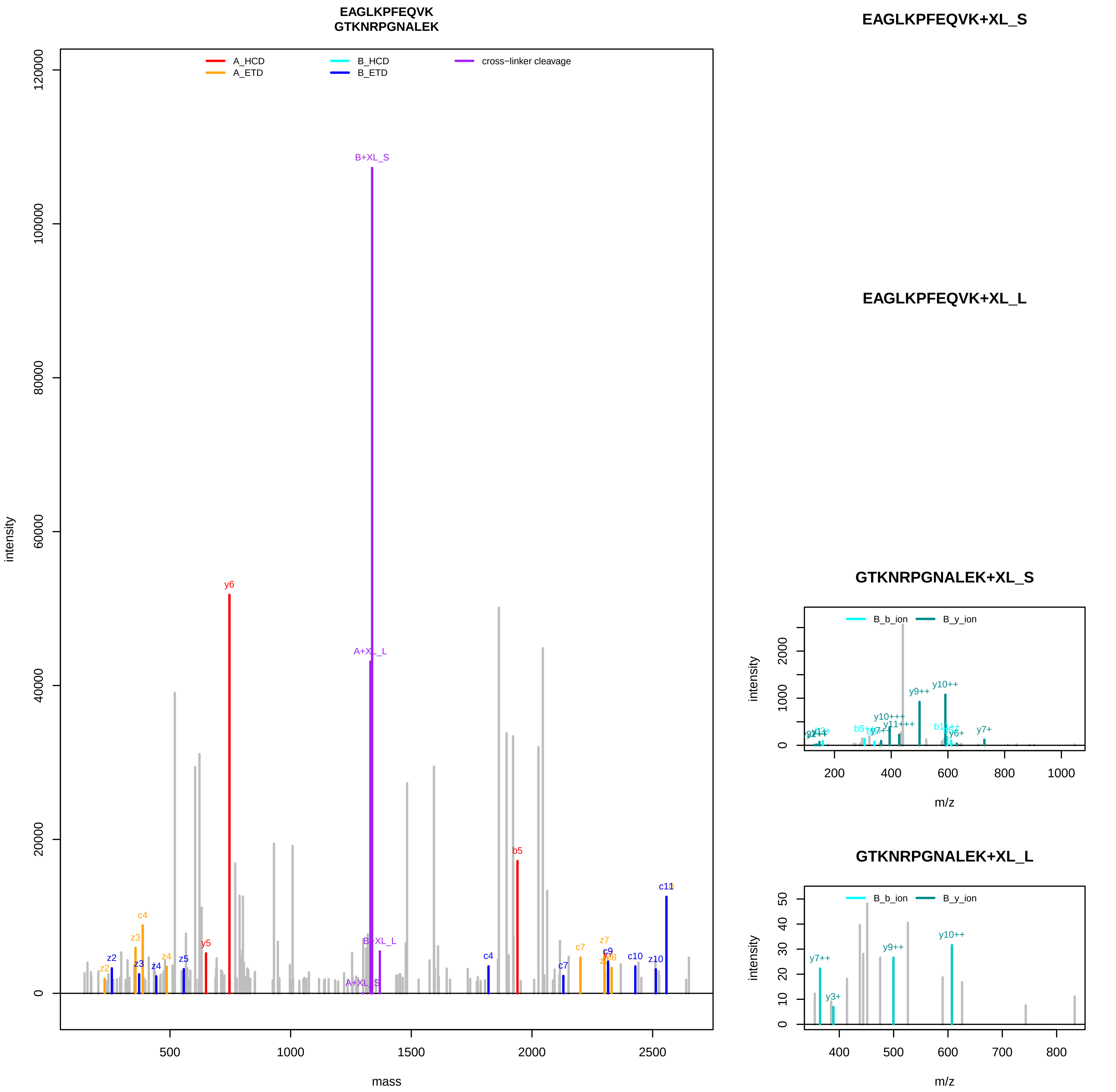
EAGLKPFEQVK
GTKNRPGNALEK
A_HCD
A_ETD
B_HCD
B_ETD
cross−linker cleavage
B+XL_S
A+XL_L
B+XL_L
A+XL_S
y6
y5
b5
c4
z3
z2
z4
z5
c7
z7
c9
c10
z10
c11
intensity
mass
EAGLKPFEQVK+XL_S
EAGLKPFEQVK+XL_L
GTKNRPGNALEK+XL_S
B_b_ion
B_y_ion
y9++
y10++
y10+++
y11+++
y7+
y6+
m/z
GTKNRPGNALEK+XL_L
y7++
y3+
y9++
y10++
